# Library‐on‐Library Intercellular Labeling for Selection of Biotin Ligase and Acceptor Peptides

**DOI:** 10.1002/cbic.202500804

**Published:** 2026-01-09

**Authors:** Benya Lakkanasirorat, Phatipon Kongkamnead, Rawiporn Amornloetwattana, Pansa Leejareon, Chayasith Uttamapinant, Wenjing Wang

**Affiliations:** ^1^ School of Biomolecular Science and Engineering Vidyasirimedhi Institute of Science and Technology (VISTEC) 555 Moo 1 Payupnai Wangchan, Rayong 21210 Thailand; ^2^ Life Sciences Institute University of Michigan 210 Washtenaw Avenue Ann Arbor MI 48109 USA; ^3^ Department of Chemistry University of Michigan 930 N University Avenue Ann Arbor MI 48109 USA

**Keywords:** acceptor peptides, biotin ligase, *cis*‐labeling, library‐on‐library selections, *trans*‐labeling

## Abstract

Library‐on‐library (LOL) selection screens combinatorial libraries to generate new protein pairs. Previously, LOL selection has only been applied to stable protein–protein interactions. To extend LOL to transient enzyme–substrate pairs, a generalizable sequential LOL *trans*‐ and *cis*‐labeling platform is developed, and a proof‐of‐concept selection is performed on *Escherichia coli* biotin ligase (BirA) and its acceptor peptide (AP). Using yeast surface display, AP mutant libraries are selected against BirA mutant libraries to identify AP variants *trans*‐biotinylated by BirA mutants. Matched BirA mutants are subsequently enriched via the SpyTag–SpyCatcher‐mediated *cis*‐labeling platform. This represents the first demonstration of enzyme–peptide substrate LOL selection and offers a versatile framework for engineering new enzyme–peptide substrate pairs with varied activities.

## Graphical abstract

The library‐on‐library selection platform first identifies novel target peptides via intercellular *trans*‐biotinylation, followed by the selection of matched enzyme variants through SpyTag–SpyCatcher‐mediated *cis*‐biotinylation. Using *Escherichia coli* biotin ligase and its acceptor peptide as a model system for site‐specific conjugation, this method provides a proof of principle for the coevolution of enzyme–peptide pairs with tunable activity.

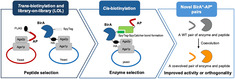

## 1 Introduction

Selection of complementary protein pairs from combinatorial libraries can significantly accelerate the development of new or improved protein–protein interactions.^[^ ^1^ ^]^ Such “library‐on‐library” (LOL) approaches have been successfully applied to Z‐domain‐affibody pairs,^[^ ^1^ ^]^ antigen identification for given T‐cell receptors (TCRs) and TCR‐peptide major histocompatibility complex interactions,^[^ ^2^ ^]^ and single‐chain variable fragment binders for HIV‐1 gp160 protein fragments.^[^ ^3^ ^]^ Current robust LOL designs are either based on the use of surface display in solely yeast^[^ ^2^ ^]^ or a combined phage‐yeast system^[^ ^3^ ^]^ to present two partner libraries on different cell populations, or two partner libraries connected in *cis* on the same cell surface.^[^ ^1^ ^]^ These approaches leverage the large library sizes provided by phage and yeast hosts, along with the ability to assess the strength of selected interactions using fluorescence‐activated cell sorting (FACS). Matched binder pairs can then be identified through sequencing, linking binding function to sequence information.

We aim to extend the LOL approach to engineer enzymes with multiple turnover capabilities. Unlike previous schemes, which rely on trapping interactions, we are interested in developing a generalizable approach targeting enzymes that interact transiently with their protein or peptide substrates, without forming stable complexes. Such a scheme could potentially be applied to engineer nontrapping split enzyme systems,^[^ ^4^, ^5^, ^6^, ^7^ ^]^ where both fragments are simultaneously optimized during the evolution, and enzymes that act on protein or peptide substrates for site‐specific labeling applications.^[^ ^8^, ^9^, ^10^ ^]^ Among these enzymatic activities, enzyme‐mediated labeling of peptide tags is widely employed for site‐specific protein modifications, visualization, and pull‐down assays in biochemical studies. Common enzyme–tag pairs include *Staphylococcus aureus* Sortase A‐mediated transpeptidation,^[^ ^11^ ^]^ *Escherichia coli* lipoic acid ligase (LplA) and its acceptor peptide (LAP),^[^ ^12^ ^,^ ^13^ ^]^ and *E. coli* biotin ligase (BirA) and its 15‐residue acceptor peptide (AP or AviTag).^[^ ^14^ ^]^ To date, directed evolution efforts have focused on improving either enzyme's labeling efficiency^[^ ^15^ ^]^ or the tag's sequence and orthogonality,^[^ ^16^ ^]^ but typically in isolation.

Here, we present a generalizable LOL platform for enzyme–tag pairs, using BirA–AP as a proof of principle. *E. coli* BirA is an ATP‐dependent enzyme that catalyzes the covalent attachment of biotin to a specific lysine residue on either its natural protein substrate BCCP or the engineered AP peptide. BirA‐catalyzed biotinylation of AP‐tagged proteins can utilize the strong biotin–streptavidin interaction to attach probes with site‐specificity for numerous applications, including imaging, immobilization, and affinity purification. BirA has been engineered to alter its small‐molecule substrate specificity to be beyond biotin,^[^ ^17^ ^,^ ^18^ ^]^ and has been converted into a proximity labeling enzyme for spatially restricted tagging of proteins.^[^ ^19^ ^,^ ^20^ ^]^ However, alternative BirA–AP tag pairs are much less investigated beyond the development of an orthogonal BirA–AP pair from yeast.^[^ ^21^ ^]^ Additional BirA–AP pairs with tunable reaction kinetics are potentially useful for interaction‐dependent labeling of protein–protein interactions with different levels of affinity.^[^ ^22^ ^]^ Therefore, we developed a LOL *trans*‐labeling and subsequent *cis*‐labeling selection platform and applied it to select new BirA–AP tags.

In this study, we first validated *trans*‐biotinylation of AP by wild‐type (WT) BirA and applied this assay to select AP peptide variants for WT BirA. Next, we performed AP library‐against‐BirA library selection to identify new AP variants that can be biotinylated by the BirA library variants. We then developed a *cis*‐biotinylation strategy on the yeast surface by leveraging SpyTag–SpyCatcher covalent capture to present target AP peptides to the BirA library, enabling identification of BirA variants specific for the new AP variants. We characterized the resulting BirA–AP pairs on yeast cells using both *trans*‐ and *cis*‐biotinylation assays and in vitro using high‐performance liquid chromatography (HPLC)‐based assays with synthetic AP peptides and purified BirA variants. To our knowledge, this is the first demonstration of enzyme–peptide tag LOL selection, and it provides a versatile framework for evolving enzyme–peptide substrate pairs with varied activities.

## 2 Results

### 2.1 Establishing *T* *rans*‐Biotinylation of BirA Against AP Peptide on the Yeast Surface

To facilitate BirA library‐against AP peptide library selection, we used yeast cell surface display‐based directed evolution, as it allows screening of millions of variants, multicolor characterization, and discrimination across a wide dynamic range of activity. Since enzyme–substrate pairs typically exhibit much weaker affinities than stable protein–protein interactions, an alternative screening strategy is required to adapt yeast surface display‐based LOL selection for enzyme–substrate LOL selection.

**Figure 1:**
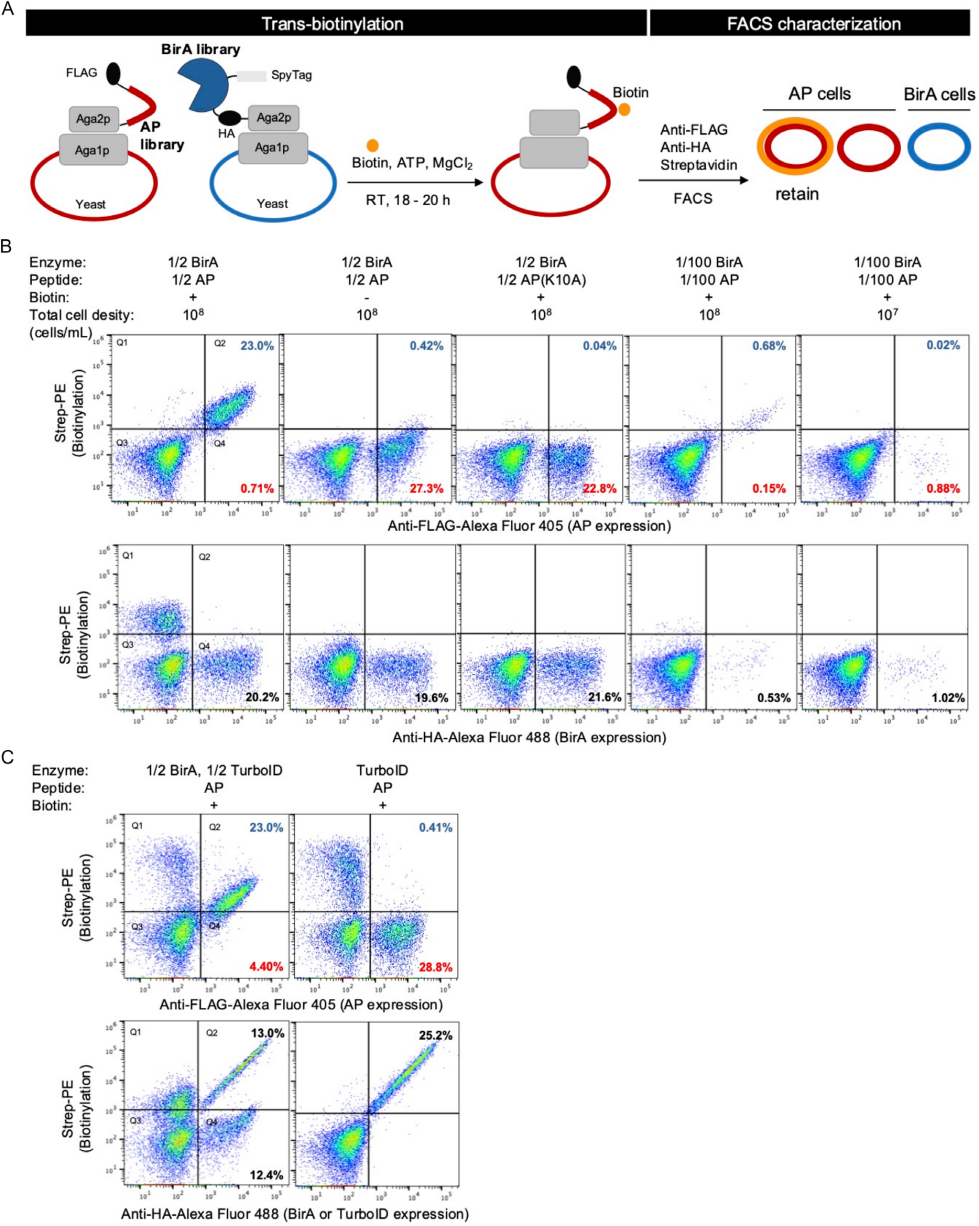
The *trans*‐biotinylation platform for a LOL selection of BirA–AP pairs. A) Schematic of *trans*‐biotinylation on the yeast cell surface for AP library‐on‐BirA library selection using FACS analysis and sorting. The AP library with a FLAG epitope tag and the BirA library with a HA epitope tag are surface‐displayed on separate yeast populations. Biotin, ATP, and MgCl_2_ were added to the mixture of AP‐displaying cells and BirA‐displaying cells and incubated at room temperature for 18–20 h. Expression of BirA, AP, and the extent of biotinylation on AP were detected by anti‐HA staining, anti‐FLAG staining, and streptavidin–phycoerythin, respectively. AP cells with high biotinylation signals were subsequently enriched by FACS. B) Characterization of *trans*‐biotinylation by BirA cells on AP cells. Top plots: biotinylation against AP expression; bottom plots: biotinylation against BirA expression. The percentages of cells in Q2 and Q4 were calculated over the total cell population, representing biotin‐positive and biotin‐negative AP‐ or BirA‐expressing cells, respectively. In column 1–3, BirA and AP cells were mixed at a 1:1 ratio, with the total cell density of 10^8 ^cells mL^–1^. In column 4, BirA cells and AP cells were present at a 1:1 ratio but further diluted 100‐fold compared to column 1–3. In column 5, BirA cells and AP cells were present at a 1:1 ratio, diluted 100‐fold compared to column 1–3, and had a 10‐fold lower total cell density compared to column 1–4. C) Characterization of *trans*‐biotinylation with BirA‐, TurboID‐, and AP‐ expressing cells. The percentages displayed are the same as in (B).

To enable LOL selection of enzyme–substrate pairs in a multiturnover reaction setup, we employed *trans*‐biotinylation in the initial screening. To validate *trans*‐biotinylation in the yeast surface display system, we constructed an aga2p fusion with BirA followed by an HA epitope tag and an aga2p fusion with AP peptide followed by a FLAG epitope tag (**Figure** **1**A). We first tested mixing BirA‐displaying yeast and AP‐displaying yeast in a 1:1 ratio with a final cell density of around 10^8 ^cells mL^–1^. In the presence of biotin, the AP‐expressing cells, but not the BirA‐expressing cells, exhibited strong biotinylation signal (Figure 1B, first column). In the negative controls without biotin supplementation or with the lysine‐null inactive AP (K10A), the AP‐displaying cells showed little biotinylation (Figure 1B, second and third columns). This suggests *trans*‐biotinylation could take place and that biotinylation is specific to the acceptor lysine residue (K10) in the AP peptide.

To better mimic the LOL *trans*‐labeling scenario where the density of cells containing a particular AP peptide or BirA variant would be much smaller, we added only 1% of each of the BirA‐displaying and AP‐displaying cells; the other 99% of cells were uninduced yeast cells, making up the final cell density of 10^8 ^cells mL^–1^ for *trans*‐biotinylation labeling. Even in this diluted cell density setup, 81% of the AP‐positive cells still exhibited biotinylation (Figure 1B, fourth column), demonstrating *trans*‐biotinylation is still effective with the low density of AP‐ and BirA‐positive cells. In contrast, AP‐expressing cells did not show detectable biotinylation at a reduced cell density of 10^7 ^cells mL^–1^ (Figure 1B, fifth column and S1), suggesting when the positive cell density is below certain level, *trans*‐biotinylation is no longer possible.

To further validate the feasibility of the LOL *trans*‐labeling selection platform, we evaluated the *trans*‐biotinylation labeling efficiency by promiscuous BirA. Even though WT BirA is highly specific for the AP peptide, mutations could cause BirA to have reduced affinity toward biotin‐AMP, resulting in inadvertent release of the reactive intermediate, which could label proximal proteins and cause false positives in the selection for the BirA and AP mutants. We displayed TurboID,^[^ ^19^ ^]^ a highly active promiscuous BirA, on yeast surface and performed *trans*‐labeling with AP‐displayed yeast. We observed that only 0.41% of the total cell population (and 1.4% of the AP‐displaying cells) showed biotin signal, while the remaining 28.8% of the total cell population did not have biotin labeling (Figure 1C, right). TurboID‐positive cells further exhibited strong biotinylation due to proximity labeling largely contained at the surface of enzyme‐expressing cells. These results suggested that promiscuous BirA as exemplified by TurboID does not *trans*‐label neighboring cells in the conditions tested. We also mixed BirA, TurboID, and AP‐displaying yeast all together. As expected, the AP‐positive cells showed strong biotinylation in the presence of BirA (Figure 1C, left). These results suggest that, under the conditions tested, promiscuous BirA variants such as TurboID do not efficiently *trans*‐biotinylate neighboring AP cells, supporting the specificity of the LOL *trans*‐labeling screening approach.

### 2.2 AP and BirA Library Construction

**Figure 2:**
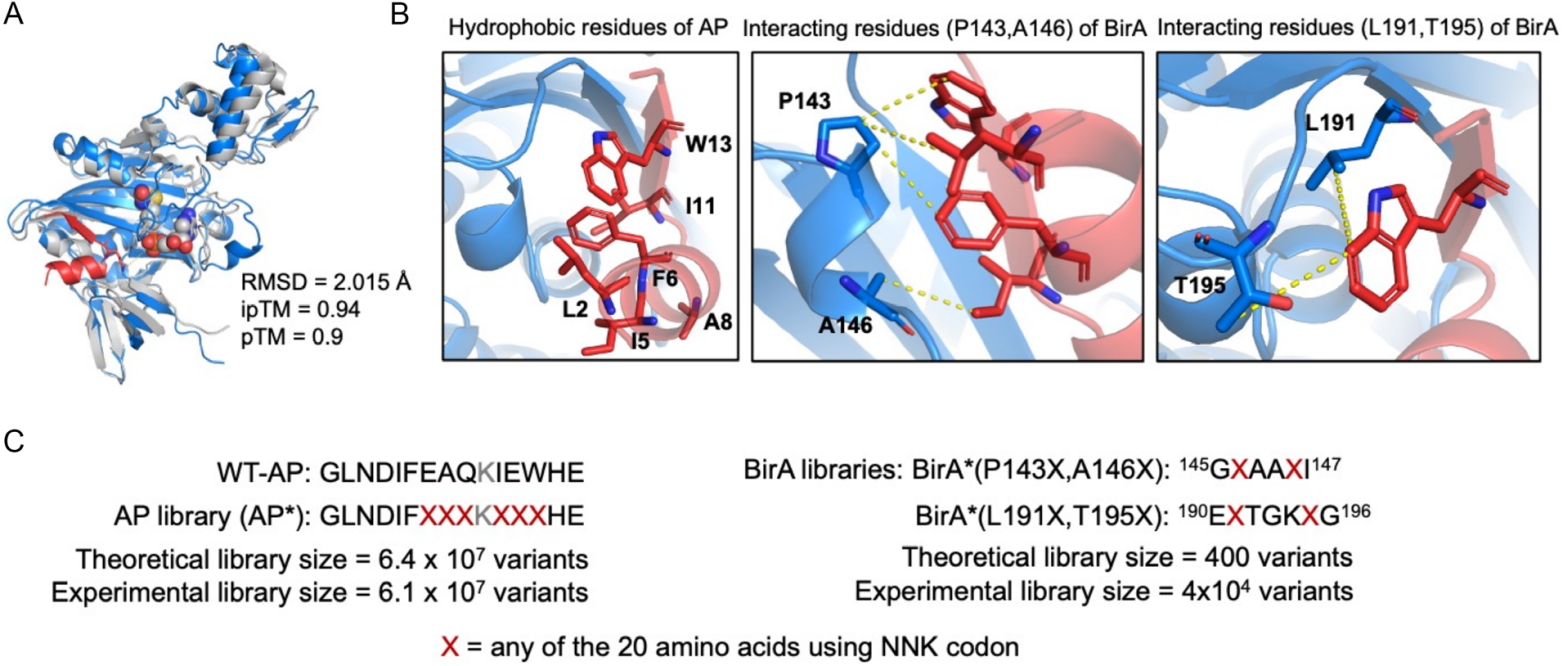
Structural analysis of the predicted BirA–AP complex and library generation. A) Alignment of the crystal structure of *E. coli* BirA (PDB: 2EWN, gray) and the predicted structure of the BirA–AP complex (BirA in blue; AP in red). Root mean square deviation (RMSD) was calculated for the alignment of all atoms. Predicted template modeling score (pTM) and interface pTM score (ipTM) are ranged from 0 to 1 and are matrices for the quality of the predicted complex structure. B) Left, close‐up views of hydrophobic residues of AP (in red) which interact with BirA (in blue). BirA residues P143 and A146 (middle), and L191 and T195 (right) which interact with AP are highlighted. The yellow dash lines indicate possible hydrophobic interactions between residues based on distance. C) AP and BirA library construction with degenerate codons. For the AP library, the six residues surrounding the acceptor lysine K10 (E7, A8, Q9, I11, E12, and W13) were selected for mutagenesis. For the BirA libraries, the two sets of interacting residues: 1) P143 and A146 and 2) L191 and T195 were selected to construct two separate libraries. All mutated residues were generated by NNK degenerate primers and theoretical and experimental library sizes were shown.

For AP library construction, we randomized the six amino‐acid residues (E7, A8, Q9, I11, E12, and W13) directly flanking the K10 acceptor lysine. Randomizing six residues with degenerate NNK codons produces AP variants at levels approaching the transformation efficiency limit of yeast. We targeted these residues because residues surrounding the acceptor lysine are highly conserved for the natural substrate across evolutionary boundaries^[^ ^23^ ^]^ and likely critical for BirA interaction based on the structural prediction of BirA–AP interactions (**Figure** **2**A). The six positions were fully randomized with the NNK degenerate codon, resulting in a theoretical library size closely matching the experimental library size of 6.1 × 10^7^ mutants (Figure 2C).

In contrast to the large AP* library, we decided to construct more focused BirA libraries with only two residues randomized. This ensures each BirA library variant was represented at close to the 1% density in 10^8 ^cells mL^–1^ that we had previously established. Based on the BirA–AP complex structure predicted by AlphaFold3 (Figure 2A,B), the highlighted hydrophobic residues in AP are likely critical for its interaction with BirA (Figure 2B, left). We then identified two sets of two BirA residues that are in proximity to the hydrophobic residues in AP for BirA library construction (Figure 2B, middle and right). The library randomized at positions P143 and A146 was designated library 1, and the library randomized at positions L191 and T195 was designated library 2, with each library containing up to 400 variants (Figure 2C).

### 2.3 AP Library *T* *rans*‐Biotinylation Selection Against WT BirA

**Figure 3:**
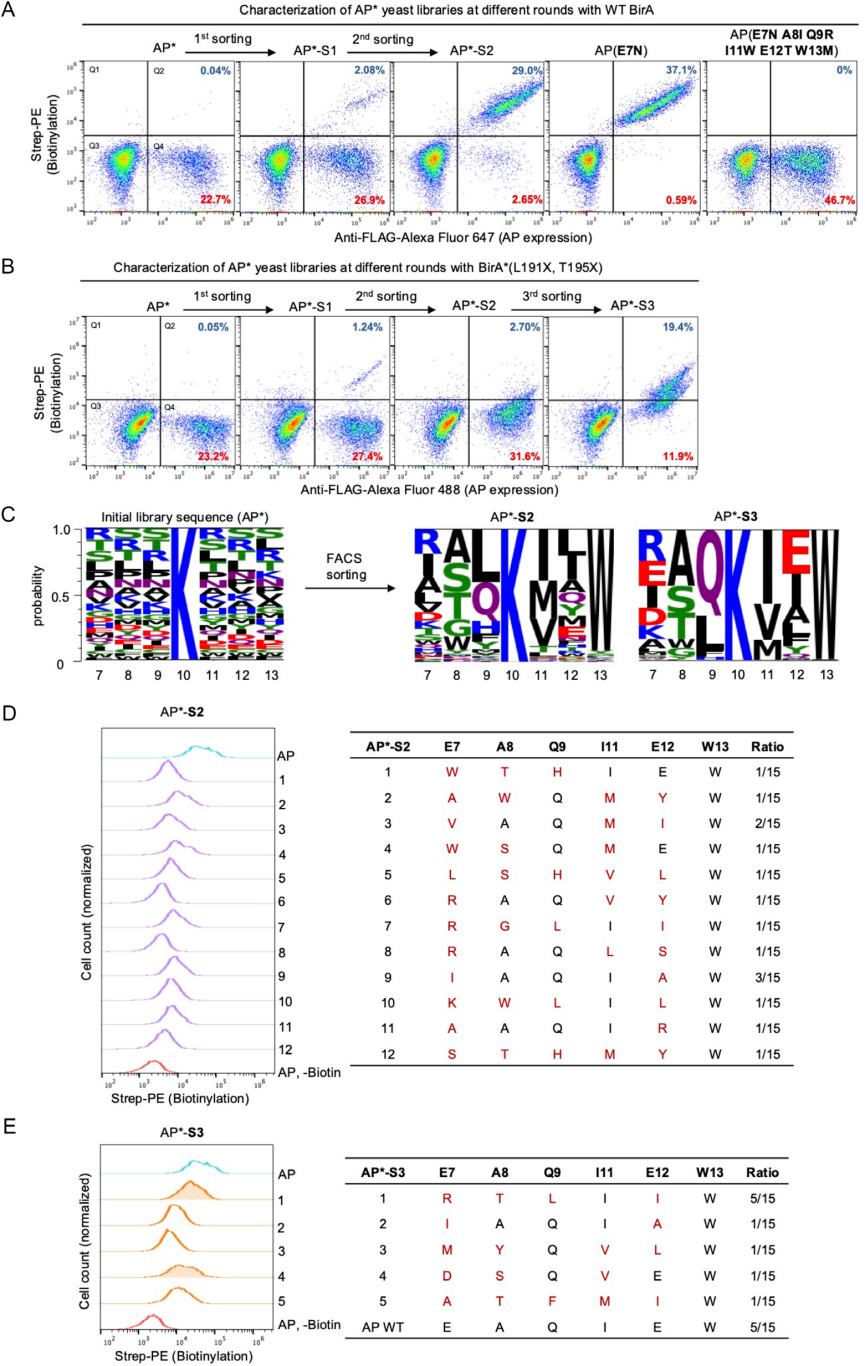
*Trans*‐biotinylation of BirA library‐on‐AP library and selection of active AP variants. A) FACS characterization of the AP* library selection against WT BirA. The percentages of cells in the Q2 and Q4 were calculated over the total cell population, representing biotin‐positive and biotin‐negative AP‐expressing cells, respectively. AP* represents the initial AP library; AP*‐S1 is the population after the first round of selection; AP*‐S2 is the population after the second round of selection; AP(**E7N**) and AP(**E7N, A8I, Q9R, I11W, E12T, W13M**) are the final AP mutants identified after selection. B) FACS characterization of the AP* library selection against BirA*(L191X T195X) library. The percentages displayed are the same as in (A). C) Sequence representation of the initial AP* library and the postsorting AP* (AP*‐S2 and AP*‐S3). D,E) Characterization of the biotinylation activity by the BirA*(L191X T195X) library on the individual AP* clones identified in the (D) AP*‐S2 population and (E) AP*‐S3 population.

We first performed AP library (AP*) selection against WT BirA to demonstrate the *trans*‐biotinylation selection platform. As shown in **Figure** **3**A, a gate was drawn to select the AP‐ and biotinylation‐positive population (Figure S2, Supporting Information). After one round of selection, the AP‐ and biotinylation‐positive population increased significantly, accounting for 7.2% of the AP‐positive cells. We repeated the selection two more times until 92% of the AP‐positive cells were biotinylation‐positive. The DNA from the enriched yeast cells was extracted for sequencing analysis. While the majority of enriched cells comprised WT AP, two unique AP* variants were also identified. The AP*(E7N) mutant exhibited strong biotinylation by WT BirA, comparable to the WT AP levels, whereas the AP*(**E7N A8I Q9R I11W E12T W13M**) mutant showed no biotinylation with WT BirA (Figure 3B and S3, Supporting Information). These findings further validated the feasibility of the yeast surface display‐based LOL *trans*‐biotinylation selection platform.

### 2.4 AP Library *T* *rans*‐Biotinylation Selection Against BirA Library

Next, we performed LOL *trans*‐biotinylation selections of the AP library (AP*) against the two BirA libraries (BirA*). For BirA*(P143X, A146X) selection, we failed to enrich AP‐positive and biotinylation‐positive cells after the first round of sorting (Figure S4, Supporting Information) and did not follow up further with this library.

The BirA*(L191X, T195X) selection enriched the AP‐ and biotinylation‐positive population (Figure S5A and S5B, Supporting Information). Two new populations of AP‐ and biotinylation‐positive yeast emerged after one round of selection (Figure 3B, first and second column). One population showed strong biotinylation signal comparable to the WT AP–BirA signal, and the other showed lower biotinylation signal. We reasoned that the highly biotinylated population is most likely due to the biotinylation of WT AP, since WT BirA is also present in the BirA library. In contrast, the population with reduced biotinylation is more likely due to newly emerged AP*–BirA* pairs. To avoid selection of WT AP, we drew a gate to enrich only the AP‐positive yeast population with lower biotinylation signal in the second round of sorting (Figure S5B, Supporting Information). Therefore, after the second round of sorting, we removed the majority of the highly biotinylated population that resembled WT AP (Figure 3B, third column). While this strategy risked missing some highly active AP variants for the BirA mutants, it is a generally effective approach for isolating new AP variants. A third round of sorting was performed to enrich the biotinylation‐positive cells, resulting in two populations of yeast with high and medium biotinylation signal (Figure 3B, fourth column, and S5B, Supporting Information). The appearance of cell populations with distinct biotinylation levels strongly suggested new AP variants have been selected.

The DNA was extracted from the enriched yeast from the post‐second round (AP*‐S2) and post‐third round (AP*‐S3) of sorting for next‐generation sequencing (NGS) analysis. The DNA was also retransformed into bacteria for individual DNA sequencing analysis. The AP*‐S2 population showed high sequence variation with no enrichment of any specific mutants, and as expected, NGS revealed WT AP accounted for only 0.25% of the total sequence reads (Figure 3C). The removal of the majority of WT AP was encouraging and validated our approach to minimize the presence of WT AP in the library. Interestingly, position 13 strongly converged to the native tryptophan, underscoring its critical role in AP's interaction with BirA. This finding further supports our hypothesis that hydrophobic residues are essential for AP binding to BirA (Figure 2B). In contrast, the AP*‐S3 population showed two enriched AP* variants: WT AP and AP(E7R A8T Q9L E12I) (Figure 3C).

We characterized the individual clones identified via individual sequencing that matched with the NGS analysis results. We characterized 12 variants from AP*‐S2 (Figure 3D). As expected, all these variants exhibited low biotinylation signal, consistent with the sorting results in Figure 3B. We characterized five enriched variants from AP*‐S3 (Figure 3E). AP(E7R A8T Q9L E12I), the most enriched variant, showed the highest biotinylation signal, ≈0.6‐fold that of WT AP, while the next most enriched mutant, AP(E7D A8S I11V), produced 0.4‐fold biotinylation signal compared to that of WT AP (Figure 3E and S6, Supporting Information).

To identify potential AP* variants orthogonal to WT BirA, we evaluated their biotinylation by WT BirA to select those with low activity. However, WT BirA exhibited higher activity toward these variants (Figure S7A and S7B, Supporting Information), which was unsurprising given the absence of negative selection against WT BirA. We noted that the interaction between BirA and AP is hydrophobic and depends on the specific peptide structure. The two selected peptides might possess secondary structures similar to AP, allowing them to fit into the active site and be specifically recognized by WT BirA.

Therefore, we performed a negative selection of the AP*‐S3 variants against WT BirA. However, after the negative selection, the remaining AP* variants exhibited no activity toward the BirA library. This suggests that most AP* variants were also recognized by the WT BirA and were thus eliminated during the negative selection. This outcome is unsurprising as gradual evolutionary walking is often required to significantly alter enzyme selectivity; our introduction of only two mutations to the BirA library was likely insufficient to produce a significant shift from WT activity.

Here, our main goal is to demonstrate the feasibility of the AP *trans*‐biotinylation selection platform and establish the selection protocol for BirA* variants. Therefore, we moved forward with the enriched variants for further selection of BirA variants that had activity toward the most enriched AP* variants.

### 2.5 BirA Library Selection via *C* *is*‐Biotinylation Using SpyTag–SpyCatcher

**Figure 4:**
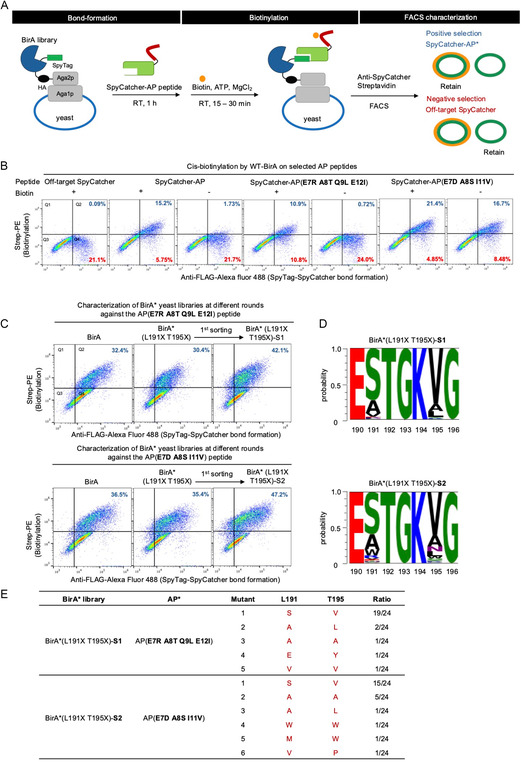
*Cis*‐biotinylation via cell‐surface SpyTag–SpyCatcher bond formation and selection of active BirA variants. A) Schematic of *cis*‐biotinylation via SpyTag–SpyCatcher bond formation on the yeast surface for the selection with BirA libraries using FACS sorting. The SpyTag‐tagged BirA library was displayed on the yeast surface. The purified SpyCatcher‐specific AP* peptide fusion protein was then incubated with BirA library cells at room temperature for 1 h. Then, biotin, ATP, and MgCl_2_ were added to initiate *cis*‐biotinylation on AP* peptides for the desired duration. SpyTag–SpyCatcher bond formation and biotinylation are detected by anti‐SpyCatcher antibody and streptavidin–phycoerythin, respectively. SpyCatcher‐positive cells with high biotinylation signals were subsequently enriched by FACS. Negative selection was performed with “off‐target” SpyCatcher with no AP fused. B) FACS characterization of *cis*‐biotinylation on AP variants using WT BirA. The percentages of cells in Q2 and Q4 were calculated over the total cell population, representing biotin‐positive and biotin‐negative SpyCatcher‐positive cells, respectively. C) FACS characterization of the BirA*(L191X T195X) library selection via *cis*‐biotinylation on the two selected AP* peptides: AP(**E7R, A8T, Q9L, E12I**) (top row); and AP(**E7D, A8S, I11V**) (bottom row). The percentages of cells in Q2 were calculated over the total cell population. BirA*(L191X T195X)‐S1 is population after the first round of selection toward AP(**E7R, A8T, Q9L, E12I**). BirA*(L191X T195X)‐S2 is population after the second round of selection toward AP(**E7D, A8S, I11V**). D) Sequence representation of the postsorting BirA*(L191X T195X)‐S1 and BirA*(L191X T195X)‐S2. E) Individual BirA* variants from the postsorting BirA*(L191X T195X) and their frequency upon sequencing of 24 clones per library.

After the new AP* were identified using the *trans*‐biotinylation platform, we next established a *cis*‐selection platform to pinpoint the BirA* variants that biotinylate specific AP* sequences. To facilitate screening of a given BirA* library with different AP* substrates, we designed the *cis*‐selection platform to be modular, with the desired AP* sequence introduced to the BirA* library‐displaying yeast in a posttranslational manner (**Figure** **4**A). To covalently conjugate AP* to the BirA*‐displaying yeast, we employed the SpyTag–SpyCatcher system.^[^ ^24^ ^]^ The 16 amino‐acid SpyTag was fused to the C‐terminus of BirA*. AP* substrates were genetically fused to SpyCatcher, expressed in *E. coli*, and purified as fusion proteins (Figure S8, Supporting Information). Addition of SpyCatcher–AP* fusion then facilitated the isopeptide bond formation between SpyTag and SpyCatcher, enabling AP* to be displayed in *cis* on the BirA*‐presenting yeast surface, and subsequent *cis*‐biotinylation. Negative selections with SpyCatcher fused to an inactive (e.g., lysine‐null) AP would be implemented to eliminate promiscuous BirA* variants that nonspecifically label proteins in proximity, independent of the presence of AP* (Figure 4A). This *cis‐*labeling selection approach enables selection of BirA* variants for specific AP peptides, like those from our initial *trans*‐biotinylation screen. However, it is not easily scalable for a library–versus–library selection because it requires the individual purification of each SpyCatcher–AP* fusion protein.

The *cis*‐biotinylation platform via SpyTag–SpyCatcher bond formation was first established using WT BirA and AP. We first confirmed successful SpyTag–SpyCatcher bond formation, as indicated by the positive correlation between the HA–BirA and SpyCatcher–AP immunofluorescence (Figure S9, Supporting Information). We then characterized the *cis*‐biotinylation efficiency by looking at the biotinylation signal versus the levels of covalently bound SpyCatcher–AP (Figure 4B, first three plots). The SpyCatcher–AP peptide exhibited high biotinylation by *cis*‐displayed WT BirA in the presence of biotin, but not in the negative controls without biotin or without AP sequence on SpyCatcher (off‐target SpyCatcher). Altogether, these experiments validated the *cis*‐labeling platform's utility for identifying BirA* variants that are active on the *cis*‐displayed AP* peptide.

We next characterized the two best SpyCatcher–AP* variants selected (Figure 4B, right four plots). Notably, we observed high biotinylation background for AP(E7D A8S I11V) in the no biotin condition. The high background of this peptide resulted from biotinylation of AP(E7D A8S I11V) by endogenous *E. coli* BirA during protein expression in *E. coli*. The high biotinylation background of AP(E7D A8S I11V) persisted when we used catalytically inactive BirA (K183A) for *cis*‐biotinylation on yeast, further confirming that the observed biotinylation occurred in steps prior to the yeast experiment (Figure S10, Supporting Information).

We then performed selection of BirA*(L191X, T195X) using *cis*‐biotinylation with the two AP* variants. Similarly, BirA* with high biotinylation signal was enriched (Figure S11, Supporting Information). After one round of sorting, the biotinylation‐positive populations with AP(E7R A8T Q9L E12I) and AP(E7D A8S I11V) increased to 42.1% and 47.2%, respectively, which were higher than the WT BirA activity levels (Figure 4C).

The DNA was extracted from the enriched yeast for NGS analysis. We observed converging mutations in BirA* sequences, with L191S and T195V mutations representing more than 50% of the total sequences. The most enriched BirA(L191S, T195V) variant is shared between the selections against the two AP* variant peptides.

### 2.6 Characterization of New BirA*–AP* Pairs

**Figure 5:**
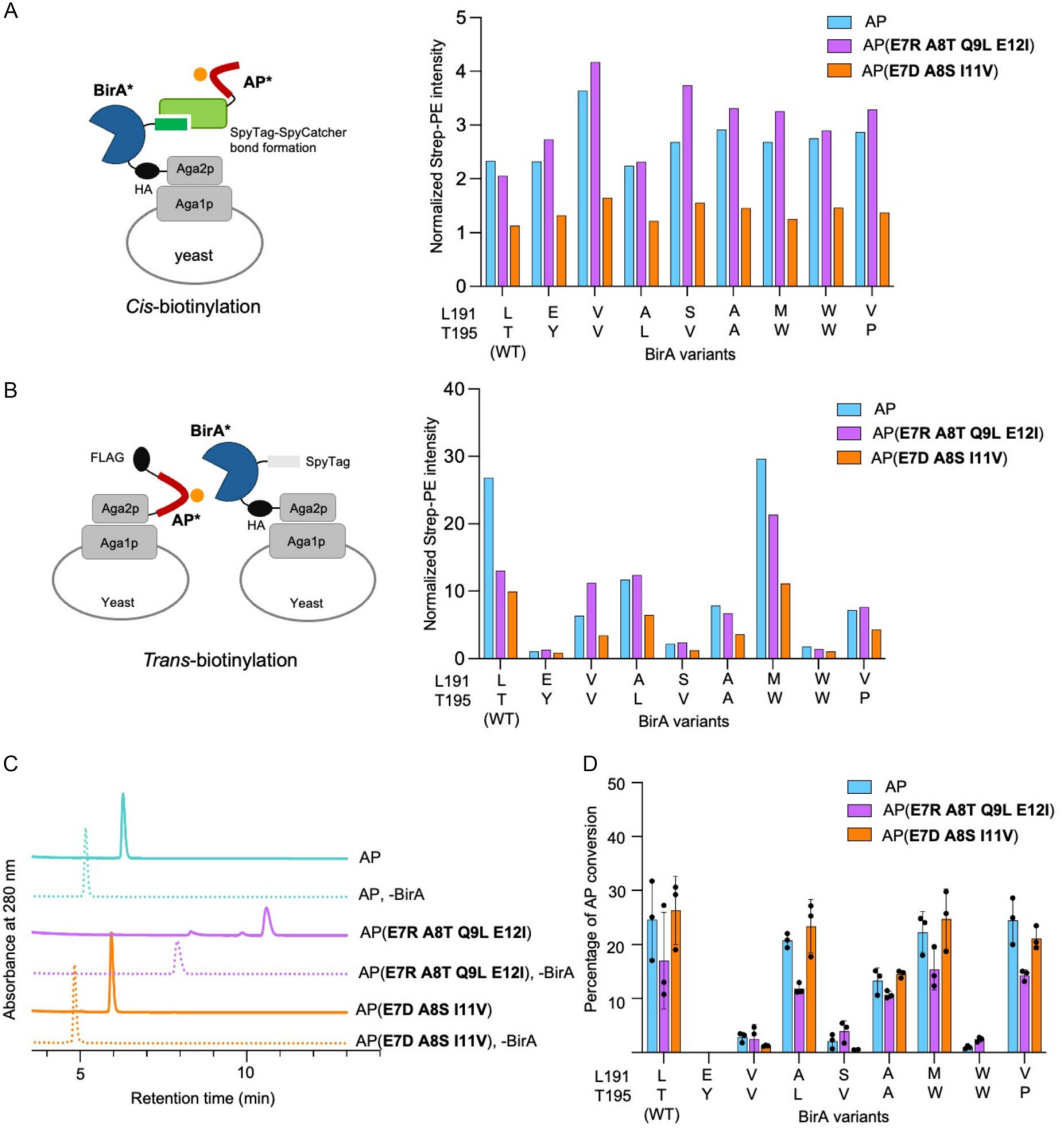
Characterization of new BirA*–AP* pairs for specific biotinylation using A) cell‐surface *cis*‐biotinylation, in which the BirA cells were incubated with 50 nM each purified SpyCatcher derivative at room temperature for 1 h, subsequently incubated with biotin, ATP, and MgCl_2_ at room temperature for 30 min; and B) *trans*‐biotinylation, in which the BirA cells and AP cells were mixed at a 1:1 ratio, with the total cell density of 10^8 ^cells mL^–^^1^, subsequently incubated with biotin, ATP, and MgCl_2_ at room temperature for 30 min. In (A) and (B), median fluorescence intensities (MFI) of biotinylation signal normalized to the negative control are shown. C) HPLC traces of in vitro biotinylation reactions (with 100 µM AP substrate) showing the distinct retention times for the AP variants (substrate) and their biotinylated products. D) Quantification of in vitro biotinylation by selected BirA* on selected AP* variants. Reactions were performed with 0.1 µM of each BirA variant for 30 min. Data are presented as mean ± SD (*n* = 3).

We then characterized the selected BirA variants via both *cis*‐ and *trans‐*biotinylation with WT AP and the two AP variants. In *cis*‐biotinylation, all the BirA variants exhibited similar *cis*‐biotinylation activity, which was at slightly higher levels than that of WT BirA with both AP* variants, but none showed a markedly different activity toward AP compared to WT BirA (**Figure** **5**A and S12A, Supporting Information). In *trans*‐biotinylation, the BirA mutants exhibited greater activity variations with both AP and AP mutants. WT BirA, BirA(L191A T195L), and BirA(L191M T195W) showed highest biotinylation signals with both AP* variants (Figure 5B and S12B, Supporting Information). Notably, BirA(L191M, T195W) produced a signal with AP(E7R, A8T, Q9L, E12I) that was 21.3‐fold higher than the negative control and 2‐fold higher than that of WT BirA. In contrast, the most enriched variant from *cis*‐biotinylation selection, BirA(L191S T195V), exhibited much lower *trans*‐biotinylation signal (Figure 5B).

The inconsistency between the *cis*‐ and *trans*‐labeling point to the limitations of the *cis*‐biotinylation approach: 1) high background due to endogenous *E. coli* biotinylation; 2) restriction to a single‐turnover reaction as BirA* and AP* are already in proximity, making selection for high turnover and catalytic efficiency challenging.

To validate the novel BirA*–AP* pairs identified from *trans*‐labeling LOL selection and subsequent *cis*‐labeling selection, we performed a comprehensive in vitro enzymatic biotinylation assay. Synthetic AP and AP mutant peptides were first biotinylated by purified BirA enzymes (Figure S13, Supporting Information) under defined reaction conditions. The reactions were subsequently quenched by EDTA and the resulting biotinylated products analyzed by HPLC. Product formation was confirmed by the appearance of a biotinylated peptide peak in a HPLC chromatogram, which exhibited a distinct retention time clearly resolved from the unreacted peptide (Figure 5C). We quantified the enzymatic efficiency by generating peptide standard curves (Figure S14, Supporting Information) and integrating the substrate (AP) and product (biotinylated AP) peak areas to calculate the percent conversion for each reaction.

The in vitro biotinylation results allowed for a quantitative comparison of biotinylation of newly evolved BirA*–AP* pairs. Both selected AP* variants were efficiently biotinylated by 0.1 µM WT BirA, yielding 17.0% and 26.3% conversion, respectively, under a 30‐min reaction (Figure 5D and S15, Supporting Information). Because the biotinylation reaction had not reached completion, these conditions were suitable for a side‐by‐side efficiency comparison of the specific BirA–AP pairs. BirA(L191A T195L), BirA(L191M T195W), and BirA(L191V T195P) all exhibited higher in vitro activities than other BirA* variants (Figure 5D). This trend was consistent with the *trans*‐, but not *cis*‐, biotinylation results on the yeast cell surface, underscoring the critical role of the *trans*‐LOL step of our selection in differentiating activities and enriching catalytically efficient BirA*–AP* pairs.

## 3 Discussion

We developed and validated a yeast surface display‐based LOL *trans*‐labeling selection platform followed by *cis*‐labeling selection, enabling the discovery of novel BirA* and AP* (BirA*–AP*) pairs. Applying selection pressure to both protein partners simultaneously in the *trans*‐labeling selection step significantly facilitated the selection of novel enzyme–substrate pairs, providing starting points for enrichment of high enzymatic activity.

As a proof of principle, we first established a *trans*‐biotinylation assay on the yeast surface, confirming biotinylation occurs specifically on AP‐expressing cells with negligible background labeling on negative controls. This platform allowed us to effectively detect the specific biotinylation when positive BirA–AP pairs were present at low frequency within large cell populations, which is a key requirement for effective high‐stringency selections in combinatorial libraries. Importantly, the platform minimizes false positives arising from promiscuous biotinylation, because the reactive intermediate biotin‐5′‐AMP has a limited diffusion radius (≈10 nm) and short half‐life once released from the BirA active site.^[^ ^25^ ^]^ As a result, the *trans*‐biotinylation approach enhances both selectivity and fidelity for the selection of new peptide substrates and corresponding BirA* variants.

A notable challenge of the LOL selection is the unintended enrichment of WT AP, since selecting for the highest AP* activity often favors WT BirA or WT BirA‐like variants. To overcome this, we performed three rounds of FACS sorting and removed enriched WT AP clones by selecting for AP* variants with moderate biotinylation. Although this strategy risks excluding peptides with WT AP activity levels, it was necessary to maintain sequence diversity in the evolving AP* pool, and helped lead to our identification of novel AP* variants with high biotinylation by BirA* variants. In future selections, the high‐activity population can also be enriched separately for sequencing analysis, which may reveal high activity, WT AP‐like mutants. NGS and individual clone analysis revealed convergent mutations at key interface residues, such as W13, consistent with structural predictions and hydrophobicity‐based recognition.

However, several selected AP* variants also showed high biotinylation activity with WT BirA, indicating persistent cross‐reactivity. For future selections that aim to achieve orthogonality, LOL selections should incorporate negative selections to select against WT activity. Additionally, computational protein design and structure‐guided library construction could refine the interface residues and further reduce WT‐like specificity. Overall, our proof of concept demonstrates that LOL selection can identify new peptide substrates tailored to specific enzyme variants.

*Cis*‐biotinylation, based on SpyTag‐mediated bond formation, enables both BirA* and AP* to be displayed on the same yeast cell surface, allowing sorting of active BirA* variants against novel peptide substrates. After a single round of FACS sorting performed separately for each of the two new peptide substrates, postsorted cells showed increased biotinylation compared to the initial BirA* library as well as WT BirA, indicating successful selection of more active BirA* variants. NGS revealed that the most common variants were shared across the two different target peptides, confirming cross‐reactivity due to the enrichment of WT‐like variants.

We observed inconsistencies in the biotinylation efficiencies of different BirA*–AP* pairs between *cis*‐ and *trans*‐biotinylation methods. While *cis*‐biotinylation in our approach allowed for feasible sorting of active BirA* variants, it has limitations, including high background signals from *E. coli* endogenous BirA during peptide expression, which can lead to saturation of biotinylation signals independent of BirA* activity and confound the positive selection. Additionally, the 1:1 stoichiometry of BirA* and AP* on the same cell permits only single‐turnover labeling, making it challenging to improve catalytic efficiency. In contrast, *trans*‐biotinylation relies on cell–cell contact for peptide substrate biotinylation and proves more effective for detecting activity in newly evolved BirA*–AP* pairs. Two potential improvements in the *cis‐*labeling scheme include: first, placing BirA*–SpyCatcher fusion protein on the yeast surface, allowing usage of synthetic SpyTag–AP* peptides to avoid prebiotinylation during *E. coli* preparation. Second, to address the single‐turnover limitation of the current *cis*‐labeling scheme, one could fuse SpyCatcher to Aga1p and introduce multiple copies of AP* fused to SpyTag.

Despite technical improvements, complete evolutionary divergence and restricted orthogonality between new BirA* and AP* pairs remain challenging. Selected variants often retain cross‐reactivity with WT partners, emphasizing the complexity of evolving protein specificity in highly conserved systems.^[^ ^26^ ^]^

Our LOL *trans*‐ and *cis‐*labeling selection platform could be broadly versatile beyond enzyme–peptide tag discovery. This approach could be applicable for engineering orthogonal protein–protein interactions and receptor–ligand pairs for synthetic biology. For example, in yeast synthetic signaling circuits,^[^ ^27^ ^]^ LOL selection can enable the creation of signaling pathways by generating matched receptors and signaling ligands with tailored specificity. Orthogonal synthetic gene regulators enable construction of programmable, insulated signal transduction networks, allowing simultaneous deployment of multiple synthetic circuits in the same cell,^[^ ^28^ ^]^ and minimize crosstalk with endogenous systems.^[^ ^29^ ^]^ Although our LOL selections yielded modest functional gains, they demonstrate the feasibility of this strategy and lay the groundwork for developing increasingly complex, orthogonal molecular tools for next‐generation synthetic biology.

## Conflict of Interest

The authors declare no conflict of interest.

## Supporting information

Supplementary Material

## Data Availability

The data that support the findings of this study are available within the article and its Supporting Information.
