# Supplementary material for: Library‐on‐Library Intercellular Labeling for Selection of Biotin Ligase and Acceptor Peptides
Source: Chembiochem. 2026 Jan 9;27(1):e202500804. doi: 10.1002/cbic.202500804 (PMC12789891; doi:10.1002/cbic.202500804)
Supplement: Supplementary file 1 — Supplementary Material [file CBIC-27-e202500804-s001.pdf]

Supporting Information

Library-on-Library Intercellular Labeling for Selection of Biotin Ligase and Acceptor Peptides

Benya Lakkanasirorat[1,2], Phatipon Kongkamnead[1], Rawiporn Amornloetwattana[1], Pansa Leejareon[1], Chayasith Uttamapinant[1]*, Wenjing Wang[2,3]*

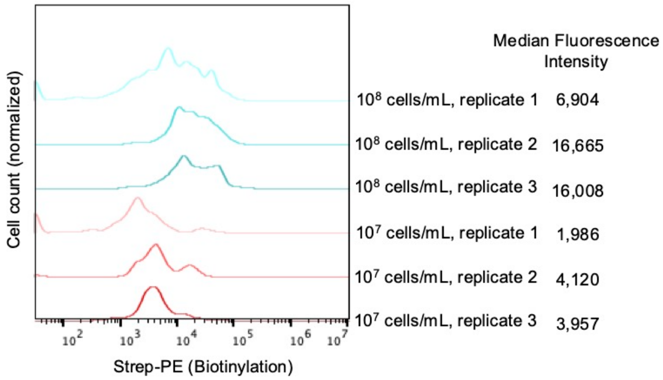

Figure S1. FACS analysis and corresponding median PE fluorescence intensities of biotinylation of AP-expressing cells at varying cell densities. Three replicates per condition are shown.

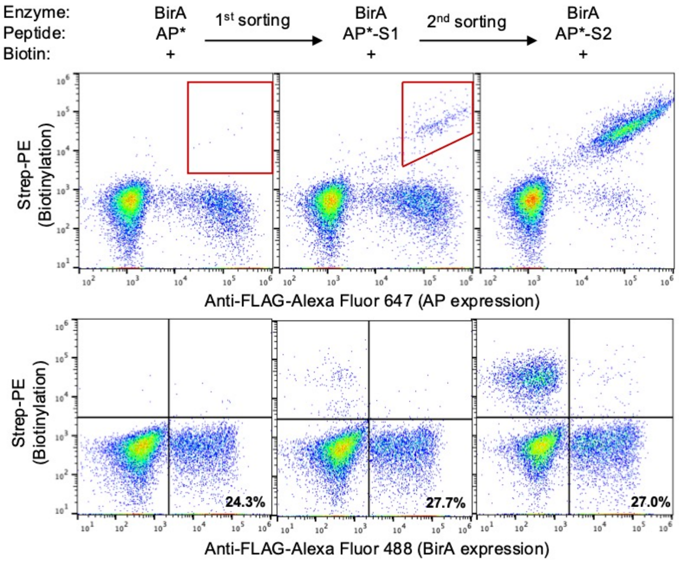

Figure S2. Gating strategy for FACS sorting of the AP* library against wild-type BirA. Top row, biotinylation against AP expression; bottom row, biotinylation against BirA expression.

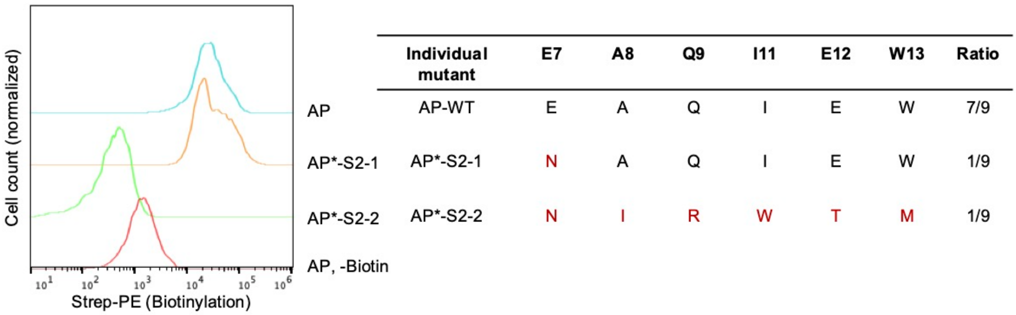

Individual mutant	E7	A8	Q9	I11	E12	W13	Ratio
AP-WT	E	A	Q	I	E	W	7/9
AP*-S2-1	N	A	Q	I	E	W	1/9
AP*-S2-2	N	I	R	W	T	M	1/9

Figure S3. Characterization of *trans*-biotinylation activity of WT BirA toward individual post-sorted AP variants (AP*-S2) by FACS analysis. Mutations in AP were identified by Sanger sequencing, and their frequencies upon sequencing of 9 clones per library are shown. Red letters highlight changed amino acids with respect to the wild-type AP sequence.

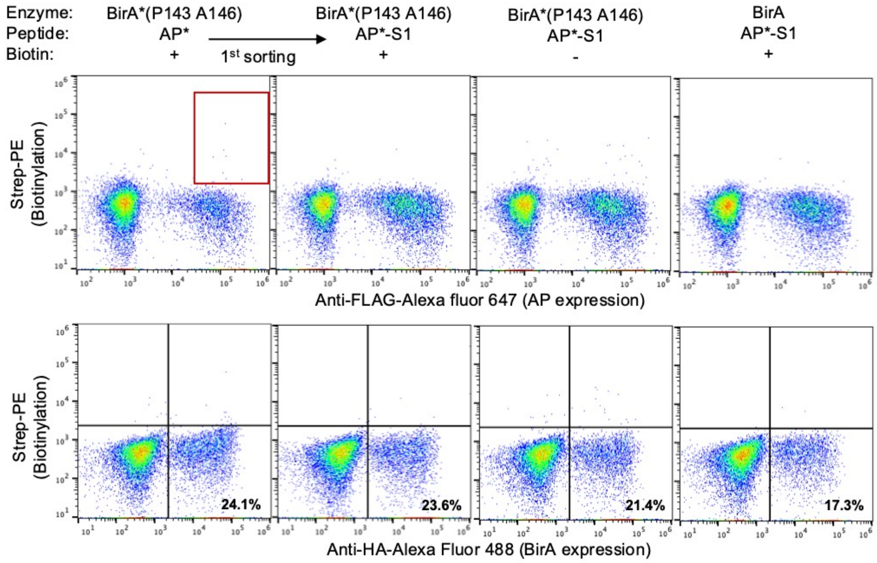

Figure S4. FACS characterization of the AP* library selection against the BirA*(P143X A146X) library. The drawn positive gate for FACS sorting is shown. Top row, biotinylation against AP expression; bottom row, biotinylation against BirA expression. The percentages of cells in the Q4 of bottom plots are calculated over the total cell population, representing BirA-positive cells. AP* represents the initial AP library; AP*-S1 is the population after the first round of selection.

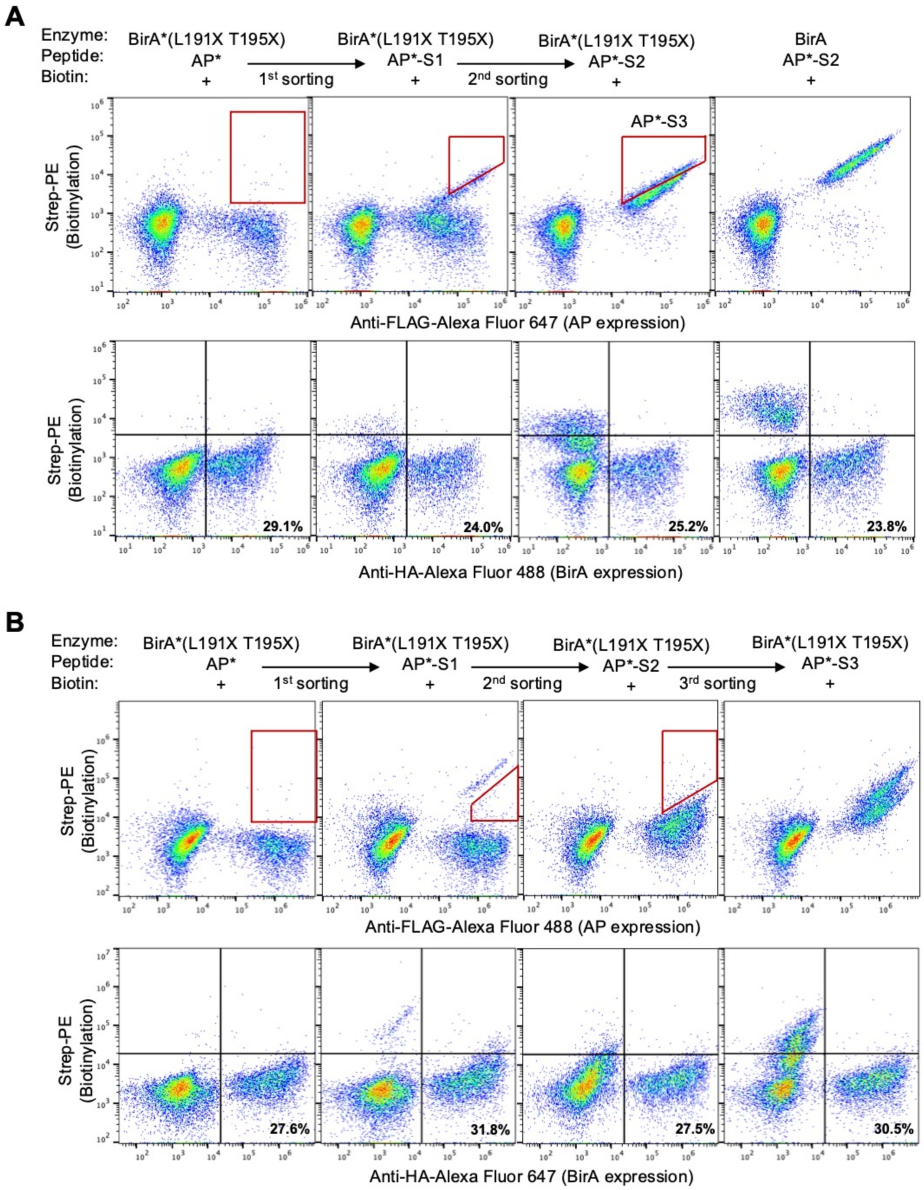

Figure S5. Gating strategy for FACS sorting of the AP* library against the BirA*(L191X T195X) library. **(A)** High biotinylation sorting gate. **(B)** Moderate biotinylation sorting gate at the second round of selection. The lower gating level was to avoid enrichment of WT AP. Top row, biotinylation against AP expression; bottom row, biotinylation against BirA expression. The percentages of cells in the Q4 of bottom plots are calculated over the total cell population, representing BirA-positive cells. AP* represents the initial AP library; AP*-S1, -S2 and -S3 are the populations after the first, second and third round of selection respectively.

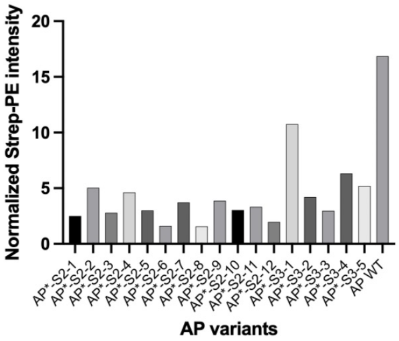

Figure S6. Characterization of the biotinylation activity by the BirA*(L191X T195X) library on the individual AP* clones. The median fluorescence intensity (MFI) of biotinylation signal was derived from the data in **Fig. 3D** and **3E.**

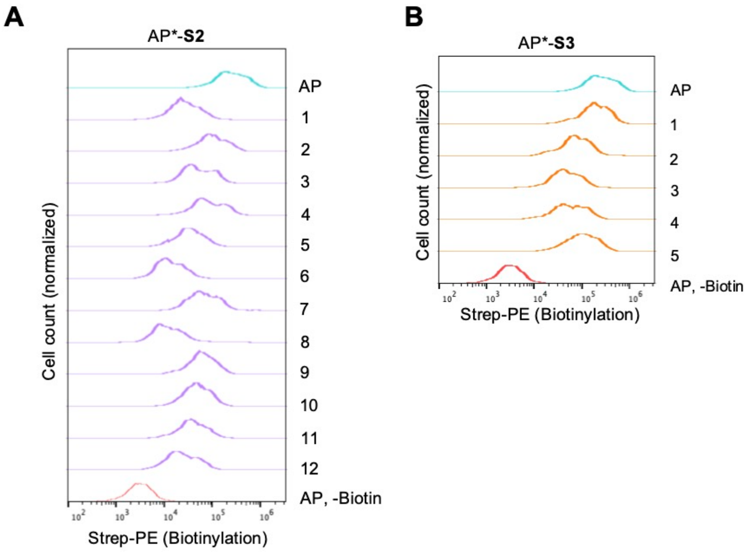

Figure S7. Characterization of the biotinylation activity by wild-type BirA on the individual AP* clones identified in the (A) AP*-S2 population and (B) AP*-S3 population. The identified variants are annotated as in Figure 3D and 3E.

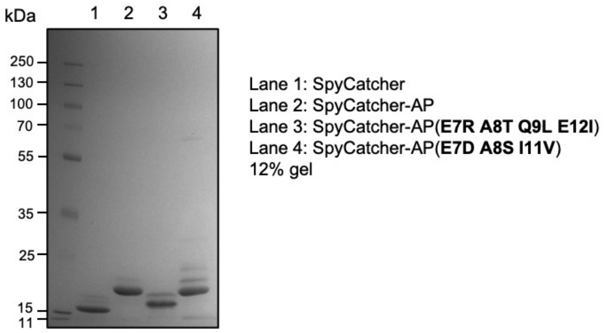

Figure S8. SDS-PAGE analysis of the purified SpyCatcher fusion proteins from *E. Coli* BL21(DE3). Net protein amount per lane, 3 μg.

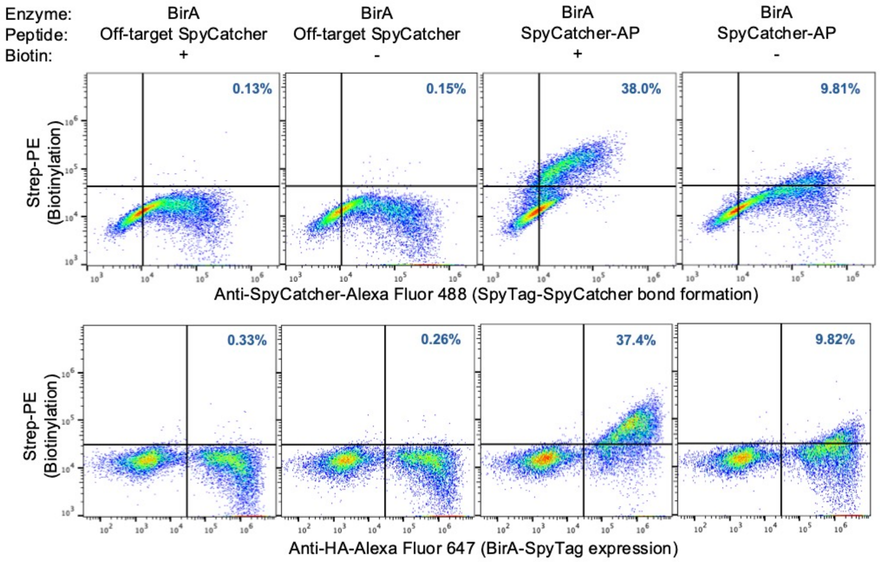

Figure S9. FACS characterization of *cis*-biotinylation on WT AP using WT BirA. Top row, biotinylation against SpyTag-SpyCatcher bond formation; bottom row, biotinylation against BirA-SpyTag expression. The percentages of cells in Q2 are calculated over the total cell population, representing biotin-positive populations on either SpyCatcher-positive cells or BirA-positive cells.

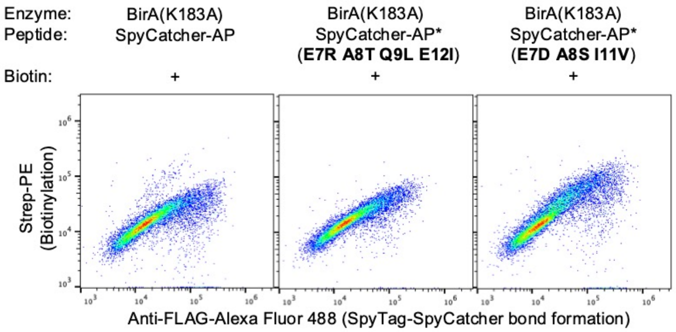

Figure S10. FACS characterization of *cis*-biotinylation on AP variants using catalytically inactive BirA(K183A). Here, the background signal from biotinylation of the SpyCatcher-AP* variant during protein expression in *E. coli* became apparent.

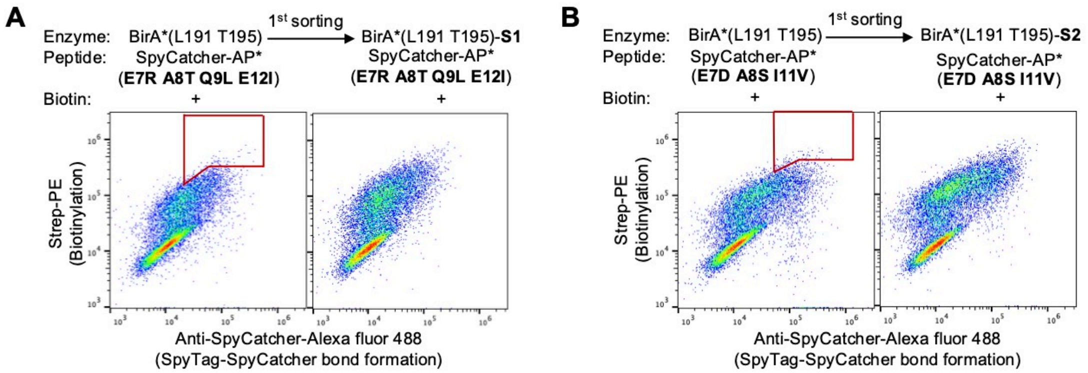

Figure S11. Gating strategy for FACS sorting of the BirA*(L191X T195X) library upon biotinylation of **(A)** AP*(**E7R A8T Q9L E12I**) and **(B)** AP*(**E7D A8S I11V**).

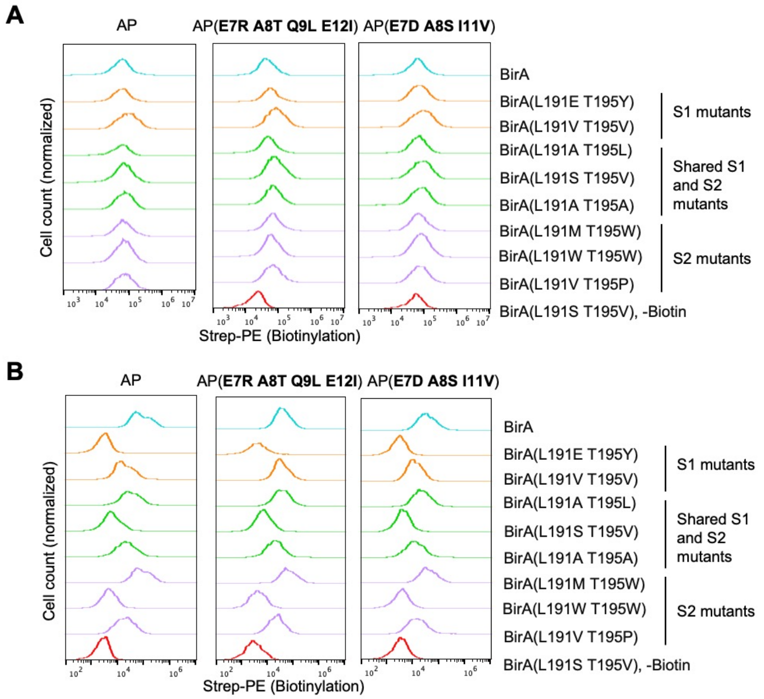

Figure S12. Characterization of new BirA*-AP* pairs for specific biotinylation. Representative histograms show **(A)** cell-surface *cis*-biotinylation and **(B)** *trans*-biotinylation assays performed under the conditions described in **Figs. 5A** and **5B**.

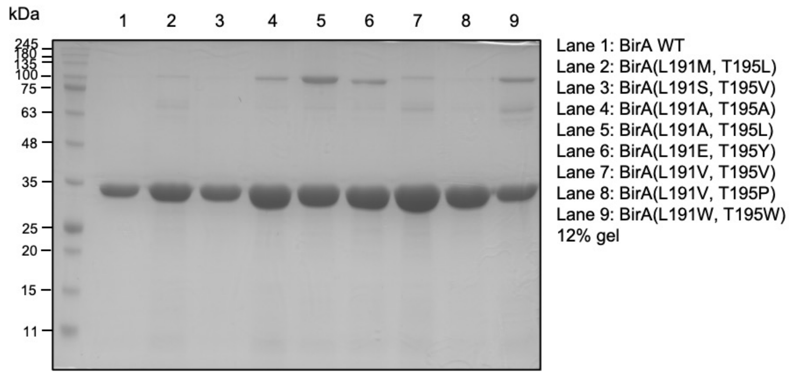

Figure S13. SDS-PAGE analysis of purified BirA variants from *E. Coli* BL21(DE3). Protein amount per lane, 5.6 µg.

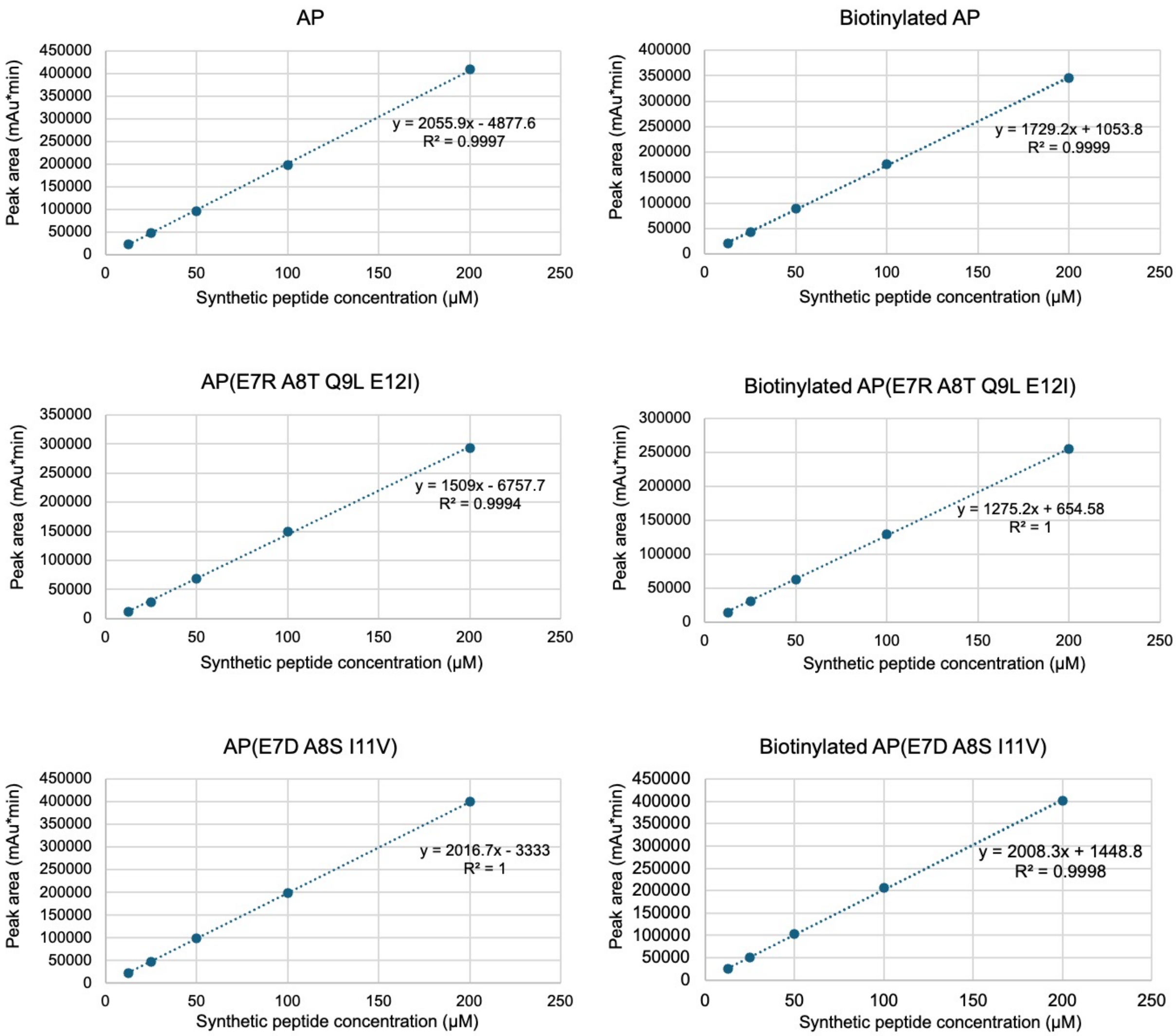

Figure S14. Standard curves for AP and biotinylated AP variants for correlation of peptide concentrations (μM) and HPLC peak areas.

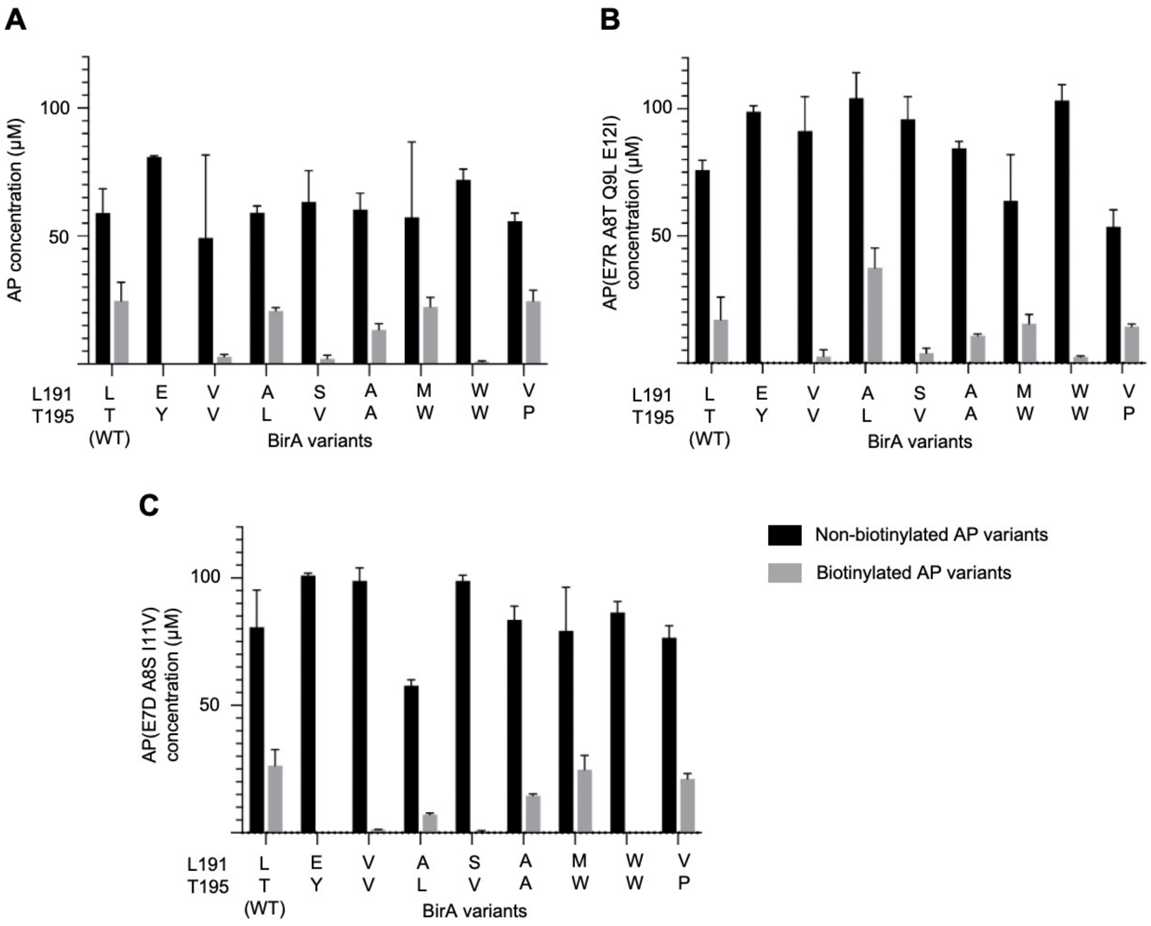

Figure S15. Quantification of substrates and products in *in vitro* biotinylation reactions. All reactions were performed with 100 μM of the respective AP variants; (A) AP, (B) AP(**E7R A8T Q9L E12I**), and (C) AP(**E7D A8S I11V**) and 0.1 μM of each BirA variant for 30 min. The concentrations were determined by HPLC analysis using standard curves (**Fig. S11**). Data are presented as mean ± SD (n=3).

Materials and Methods

Cloning

Constructs used in this study were summarized in Table for genes

For yeast display plasmids, gene fragments were amplified using Q5 polymerase (NEB) or ordered from Twist biosciences and subsequently assembled into the linearized pCTCON2 vector that was doubly digested with restriction enzymes using Gibson assembly (NEB). For protein expression plasmids, gene fragments were amplified using Q5 polymerase with primers containing mutations at specific positions to generate mutants. These were then assembled into the linearized pET21(a) vector doubly digested with restriction enzymes by Gibson assembly. The products were transformed into chemically competent *E. coli* XL1-Blue by heat shock.

AlphaFold3 prediction of the BirA-AP complex structure

Amino acid sequences of *E. coli* BirA and AP used as input for AlphaFold3 were:
BirA
>MKDNTVPLKLIALLANGEFHSGEQLGETLGMSRAAINKHIQTLRDWGVDVFTVPGKGYSLPEP
IQLLNAKQILGQLDGGSVAVLPVIDSTNQYLLDRIGELKSGDACIAEYQQAGRGRRGRKWFSPF
GANLYLSMFWRLEQGPAAAIGLSLVIGIVMAEVLRKLGADKVRVKWPNDLYLQDRKLAGILVEL
TGKTGDAAQIVIGAGINMAMRRVEESVVNQGWITLQEAGINLDRNTLAAMLIRELRAALELFEQE
GLAPYLSRWEKLDNFINRPVKLIIGDKEIFGISRGIDKQGALLLEQDGIIKPWMGGEISLRSAEK
AP
> GLNDIFEAQKIEWHE
These two sets of sequence were input into AlphaFold3 as proteins (1 copy). Structural modeling of the BirA-AP complex was performed on the AlphaFold Server. AlphaFold3 prediction provides per-residue pLDDT. Predicted template modeling score (pTM) and interface predicted template modeling score (ipTM), both numbers could range from 0 to 1, were used to evaluate the quality of predicted complex structures, with higher values indicating higher confidence in prediction. Predicted structures were analyzed and structurally aligned to the crystal structure of BirA with a bound biotin-AMP analog (PDB: 2EWN) using PyMol.

AP Library diversification and construction

AP gene were mutated using PCR with the degenerate primers containing NNK codons at the specific positions (E7, A8, Q9, I11, E12, and W13) (see Table for primers). To enhance homologous recombination between the AP fragments and the vector, an additional PCR step was performed to append overhang sequences flanking the mutated fragments. The pCTCON2 vector was doubly digested by the restriction enzymes at the EcoRI/XhoI restriction sites. A mixture of 2 μg of linearized vector and 8 μg of mutated AP fragments was concentrated using Pellet Paint Co-Precipitant (Millipore). The concentrated DNA was then electroporated into electrocompetent *Saccharomyces cerevisiae* EBY100 with Gene Pulser Xcell Electroporation System (Bio-Rad).

BirA Library diversification and construction

BirA gene were mutated using PCR with the degenerate primers containing NNK codons at the specific positions: P143 and A146 (1st library); and L191 and T195 (2nd library) (see Table for primers). Two partial BirA fragments were then assembled into the full-length mutated BirA gene using overlapping PCR. The pCTCON2 vector was doubly digested by the restriction enzymes at the NheI/XhoI restriction sites. The electroporation was performed as described for the AP library.

Yeast library culture and induction

To assess the library size, 10 µL transformed library yeast cells were serially diluted, then plated on glucose-supplemented (SD-CAA), Ura- and Trp-dropout plates and incubated at 30°C for 48 - 72 h. Numbers of colonies in each plate were counted to calculate the library size achieved. Remaining yeast cells in the recovery medium was used to inoculate 100 mL SD-CAA, Ura- and Trp-dropout medium. Yeast cells of at least 10 times yeast the library size (OD600 = 10^7 cells/mL) were sub-cultured in fresh medium to ensure that unique mutants in libraries were present during cultivation.

To induce AP or BirA expression on Aga2p, 10% of the yeast culture was inoculated in raffinose-supplemented, Ura- and Trp-dropout medium and grown for 24 h to deplete glucose. Then, 10% of this culture was transferred into galactose-supplemented (SG-CAA), Ura- and Trp-dropout medium without biotin for 20 - 24 h.

Yeast library-against-library biotinylation for AP-displaying cells and secondary labeling for FACS sorting

After inducing AP and BirA expression, AP-displaying and BirA-displaying yeasts were mixed at a 1:1 ratio, then supplemented with 50 µM biotin, 5 mM ATP, and 5 mM $MgCl_2$ in PBS-B (137 mM NaCl, 2.7 mM KCl, 10 mM Na_2HPO_4, 1.8 mM KH_2PO_4, 1% BSA). A final volume of 12 mL of ~1.0 x 10^8 / mL mixed BirA* and AP* library yeast were incubated at room temperature with rotation for desired durations in the first round of trans-labeling selection. A large volume of yeast was used to ensure sufficient coverage of the 6.1 x 10^7 AP* variants

AP expression was detected by staining cells with a mouse anti-FLAG antibody (Sigma; 1:200 dilution) at room temperature for 1 h, followed by a goat anti-mouse antibody conjugated to Alexa Fluor 647 (Life Technologies, A-21235#2369432; 1:400 dilution) or 488 (Life Technologies, A-11001#2916441; 1:400 dilution) for 1 h. BirA expression was detected by staining cells with a rabbit anti-HA antibody (Cell Signaling Technology, clone C29F4#13; 1:400 dilution) at room temperature for 1 h, followed by a goat anti-rabbit antibody conjugated to Alexa Fluor 647 (Life Technologies, A-21245#2369432; 1:400 dilution) or 488 (Life Technologies, A-11006#2282101; 1:400 dilution) for 1 h. Biotinylation of the AP-displaying cells was detected by staining with streptavidin-phycoerythrin (Life Technologies, 3052397: 1:50 dilution).

Detection of AP, BirA expression and biotinylation were often performed on the same set of cells, in which case fluorescently labeled streptavidin and secondary antibodies were added to the cells together. The yeast cell pellet was suspended in PBS-B before analysis and sorting using MA900

Multi-Application Cell Sorter (Sony Biotechnology) or BD FACSDiscover™ S8 Cell (BD Biosciences).

SpyTag-mediated AP attachment and biotinylation of BirA-displaying cells and secondary labeling for FACS sorting

After inducing BirA-SpyTag expression, 10^7 induced yeasts were incubated with 50 nM purified SpyCatcher derivative in PBS at room temperature for 1 h. Biotinylation was then performed as described in the library-against-library method.

SpyTag-SpyCatcher bond formation was detected by staining cells with a rabbit anti-SpyCatcher antibody (Bio-rad, HCA379#169426; 1:400 dilution) at room temperature for 1 h, followed by a goat anti-rabbit antibody conjugated to Alexa Fluor 488 (Life Technologies, A-11006#2282101 1:400 dilution) for 1 h. The biotinylation of the BirA-displaying cells was detected by staining with streptavidin-phycoerythrin. Detection of SpyTag-SpyCatcher bond formation and biotinylation were often performed on the same set of cells, in which case fluorescently labeled streptavidin and secondary antibodies were added to the cells together.

Next-generation sequencing

Plasmids of the post-sorted population were extracted using Zymoprep™ Yeast Plasmid Miniprep II (Zymo Research). Amplicons containing library positions were amplified by PCR for 30 cycles using primers with Illumina adapter overhangs (see Table for primers). The PCR products were purified by agarose gel purification, quantified by nanodrop, and sequenced by the Illumina MiSeq™ Series.

SpyCatcher fusion protein expression and purification

The protocol for SpyCatcher derivatives expression and purification was adapted from a previously published protocol[1]. The pET21a-based plasmid containing transgenes encoding SpyCatcher, an N-terminal His_6-tag and a C-terminal AP sequence was transformed into chemically competent *E. coli* BL21(DE3), then cultured in LB (Luria-Bertani, Miller) media with 100 mg/L ampicillin at 37 °C with shaking at 220 rpm. Saturated cultures were inoculated at 1% v/v into 1 L of LB with 100 mg/L ampicillin, then grown until OD600 ~0.5 – 0.6. Upon the culture reaching to OD600, the culture was induced with 0.42 mM isopropyl p-D-1-thiogalactopyranoside (IPTG) and incubated overnight at 16 °C with a lower shaking speed of 180 rpm. Induced cells were harvested and lysed by sonication in cold lysis buffer (50 mM Tris-HCl, 300 mM NaCl, pH = 7.8 and 1 mM phenylmethylsulfonylfluoride (PMSF)). The supernatant was then loaded onto manually packed nickel-nitrilotriacetic acid (Ni-NTA) agarose (GE healthcare) equilibrated with the Ni-NTA binding buffer (50 mM Tris-HCl, 300 mM NaCl, pH = 7.8). Impurities were washed with the washing buffer (the binding buffer + 30 mM imidazole), and proteins were subsequently eluted with the elution buffer (the binding buffer + 300 mM imidazole). Purified SpyCatcher fusion protein was concentrated and exchanged into storage buffer (PBS, pH = 7.4). The protein concentration was measured using Pierce™ 660nm Protein Assay Kit (Thermo Fisher Scientific) with a BSA standard.

BirA protein expression and purification

The pET21a-derived plasmid containing transgenes encoding BirA (WT or mutants) and a C-terminal His_6-tag was transformed into chemically competent *E. coli* BL21(DE3), then cultured in LB (Luria-Bertani, Miller) media with 100 mg/L ampicillin at 37 °C with shaking at 220 rpm. Saturated cultures were inoculated at 1% v/v into 1 L of LB with 100 mg/L ampicillin, then grown until OD_{600} reached ~0.5 – 0.6. Upon the culture reaching the desired OD_{600}, the culture was induced with 0.5 mM isopropyl p-D-1-thiogalactopyranoside (IPTG) and incubated overnight at 16 °C with a lower shaking speed of 180 rpm. Induced cells were harvested and lysed by sonication in cold lysis buffer (PBS, pH =7.8 and 1 mM phenylmethylsulfonylfluoride (PMSF)). The supernatant was then loaded onto manually packed nickel-nitrilotriacetic acid (Ni-NTA) agarose (GE healthcare) equilibrated with the Ni-NTA binding buffer (PBS, pH = 7.8). Impurities were washed with the washing buffer (the binding buffer + 40 mM imidazole), and proteins were subsequently eluted with the elution buffer (the binding buffer + 400 mM imidazole). Purified BirA protein was concentrated and exchanged into storage buffer (PBS, pH = 7.8). The protein concentration was measured using Pierce™ BCA Protein Assay Kit (Thermo Fisher Scientific) with a BSA standard.

HPLC characterization of in vitro biotinylation on synthetic AP peptides

The WT AP, AP(E7R, A8T, Q9L, E12I), AP(E7D, A8S, I11V), and negative-control AP(K10A) peptides were synthesized by GenScript. All standard curves were performed by reverse-phase HPLC using a ZORBAX Eclipse Plus C18 column with a 20-45% acetonitrile gradient (in water with 0.1% formic acid) over 13 min, with a flow rate of 1.2 mL/min, using LC-2050C I-series HPLC (Shimadzu). Typical retention times of peptide species of interest ranged from 4.8 – 10.9 min. Standard curves were prepared with varying peptide concentrations in either a non-biotinylated buffer (1 mM biotin, 4 mM ATP, and 5 mM $MgCl_2$ in 50 mM Tris, pH = 8.3) or a biotinylated buffer (non-biotinylated buffer supplemented 2 μM BirA, incubated at 30 °C for 4 h, and quenched with 50 mM ethylenediaminetetraacetic acid (EDTA)). The *in vitro* enzymatic biotinylation reactions were conducted with 100 μM AP peptide variants and 0.1 μM BirA variants in the biotinylated buffer at 30 °C for 30 min. The resulting peptide conversion was calculated by analyzing peak areas from the HPLC chromatograms against the linear regression equations derived from the peptide standard curves.

Statistical analysis

Flow cytometry data were analyzed using FlowJo v10 and are presented as median fluorescence intensity (MFI) or population percentages from representative experiments. For HPLC analysis, standard curves were quantified using linear regression. HPLC conversion rates were calculated by dividing product concentrations by the initial substrate concentration; these data are presented as mean ± standard deviation (SD) derived from three independent experiments (n=3). All data analysis and plotting were performed using GraphPad Prism.

Table for genes

Gene name	Gene sequence 5'-3'	Vector	Used in

Aga2p-AP-FLAG	ATGCAGTTACTTCGCTGTTTTTCAATATTTTCTG TTATTGCTTCAGTTTTAGCACAGGAACTGACAA CTATATGCGAGCAAATCCCCTCACCAACTTTAG AATCGACGCCGTACTCTTTGTCAACGACTACTA TTTTGGCCAACGGGAAGGCAATGCAAGGAGTT TTTGAATATTACAAATCAGTAACGTTTGTCAGTA ATTGCGGTTCTCACCCCTCAACAACTAGCAAAG GCAGCCCCATAAACACACAGTATGTTTTTAAGG ACAATAGCTCGACGATTGAAGGTAGACTGCAG GCTAGTGGTGGAGGAGGCTCTGGTGGAGGCG GTAGCGGAGGCGGAGGGTCGGCTAGCGGTTT GAATGATATTTTTGAAGCTCAGAAGATTGAATG GCATGAAGGATCCGACTACAAAGATGACGATG ACAAG	pCTCON2	Fig. 1A
Aga2p-HA-BirA-SpyTag	ATGCAGTTACTTCGCTGTTTTTCAATATTTTCTG TTATTGCTTCAGTTTTAGCACAGGAACTGACAA CTATATGCGAGCAAATCCCCTCACCAACTTTAG AATCGACGCCGTACTCTTTGTCAACGACTACTA TTTTGGCCAACGGGAAGGCAATGCAAGGAGTT TTTGAATATTACAAATCAGTAACGTTTGTCAGTA ATTGCGGTTCTCACCCCTCAACAACTAGCAAAG GCAGCCCCATAAACACACAGTATGTTTTTAAGG ACAATAGCTCGACGATTGAAGGTAGATACCCAT ACGACGTTCCAGACTACGCTCTGCAGGCTAGT GGTGGAGGAGGCTCTGGTGGAGGCGGTAGCG GAGGCGGAGGGTCGGCTAGCAAGGATAACAC CGTGCCACTGAAATTGATTGCCCTGTTAGCGAA CGGTGAATTTCACTCTGGCGAGCAGTTGGGTG AAACGCTGGGAATGAGCCGGGCGGCTATTAAT AAACACATTCAGACACTGCGTGACTGGGGCGT TGATGTCTTTACCGTTCCGGGTAAAGGATACAG CCTGCCTGAGCCTATCCAGTTACTTAATGCTAA ACAGATATTGGGTCAGCTGGATGGCGGTAGTG TAGCCGTGCTGCCTGTGATTGACTCCACGAAT CAGTACCTTCTTGATCGTATCGGAGAGCTTAAA TCGGGCGATGCTTGCATTGCAGAATACCAGCA GGCTGGCCGTGGTCGCCGGGGTCGGAAATGG TTTTCGCCTTTTGGCGCAAACTTATATTTGTCG ATGTTCTGGCGTCTGGAACAAGGCCCGGCGGC GGCGATTGGTTTAAGTCTGGTTATCGGTATCGT GATGGCGGAAGTATTACGCAAGCTGGGTGCAG ATAAAGTTCGTGTTAAATGGCCTAATGACCTCT ATCTGCAGGATCGCAAGCTGGCAGGCATTCTG GTGGAGCTGACTGGCAAAACTGGCGATGCGG CGCAAATAGTCATTGGAGCCGGGATCAACATG GCAATGCGCCGTGTTGAAGAGAGTGTCGTTAA TCAGGGGTGGATCACGCTGCAGGAAGCGGGG ATCAATCTCGATCGTAATACGTTGGCGGCCATG CTAATACGTGAATTACGTGCTGCGTTGGAACTC TTCGAACAAGAAGGATTGGCACCTTATCTGTCG CGCTGGGAAAAGCTGGATAATTTTATTAATCGC	pCTCON2	Fig. 1A and 4A

	CCAGTGAAACTTATCATTGGTGATAAAGAAATA TTTGGCATTTCACGCGGAATAGACAAACAGGG GGCTTTATTACTTGAGCAGGATGGAATAATAAA ACCCTGGATGGGCGGTGAAATATCCCTGCGTA GTGCAGAAAAAGGTGGAGGCGGATCTGGAGG TGGCGGCAGCAGAGGAGTTCCCCATATTGTTA TGGTTGATGCATATAAGAGGTATAAAGGATCCG AACAAAAGCTTATTTCTGAAGAGGACTTG		
Hisx6-SpyCatcher-AP	ATGCACCACCACCACCACCACGGTAGCGTAAC CACCTTATCAGGTTTATCAGGTGAGCAAGGTCC GTCCGGTGATATGACAACTGAAGAAGATAGTG CTACCCATATTAAATTCTCAAAACGTGATGAGG ACGGCCGTGAGTTAGCTGGTGCAACTATGGAG TTGCGTGATTCATCTGGTAAAACTATTAGTACA TGGATTTCAGATGGACATGTGAAGGATTTCTAC CTGTATCCAGGAAAATATACATTTGTCGAAACC GCAGCACCAGACGGTTATGAGGTAGCAACTCC AATTGAATTTACAGTTAATGAGGACGGTCAGGT TACTGTAGATGGTGAAGCAACTGAAGGTGACG CTCATACTGGTAGCAGCGGTAGCGGCCTGAAC GATATTTTTGAAGCGCAGAAAATTGAATGGCAT GAA	pET21(a)	Fig. 4A

Table for primers

Name	Sequence 5’-3’	Source	Used in
AP library_F	AAAAATTGTTAATATACCTCTATACTTTAACGTC AAGGAGAAAAAACCC	Sigma	Fig. 2C
AP library_R	CGTCATCTTTGTAGTCGGATCCTTCATGMNNM NNMNNCTTMNNMNNMNNAAAAATATCATTCAA ACCGCTAGCC	IDT	Fig. 2C
BirA*(P143X A146X)_F	GTTCTGGCGTCTGGAACAAGGCNNKGCGGCG NNKATTGGTTTAAGTCTGGTTATCGGTATC	IDT	Fig. 2C
BirA*(P143X A146X)_R	GCCTTGTTCCAGACGCCAGAAC	Sigma	Fig. 2C
BirA*(L191X T195X)_F	CTGGCAGGCATTCTGGTGGAGNNKACTGGCAA ANNKGGCGATGCGGCGCAAATAGTC	IDT	Fig. 2C
BirA*(L191X T195X)_R	CTCCACCAGAATGCCTGCCAG	Sigma	Fig. 2C
AP-NGS_F	ACACTCTTTCCCTACACGACGCTCTTCCGATCT CTGCAGGCTAGTGGTGGAGG	Sigma	Fig. 3C
AP-NGS_R	GACTGGAGTTCAGACGTGTGCTCTTCCGATCT GGAACGAAAAATAGAAAAGGATATTACATGGGA AAAC	Sigma	Fig. 3C
BirA(L191X T195X)-NGS_F	ACACTCTTTCCCTACACGACGCTCTTCCGATCT GTGATGGCGGAAGTATTACGCAAG	Sigma	Fig. 4D
BirA(L191X T195X)-NGS_R	GACTGGAGTTCAGACGTGTGCTCTTCCGATCT CTGATTAACGACACTCTCTTCAAC	Sigma	Fig. 4D

References

1 Keeble, A. H. *et al.* Approaching infinite affinity through engineering of peptide-protein interaction. *Proc Natl Acad Sci U S A* **116**, 26523-26533, doi:10.1073/pnas.1909653116 (2019).
